# How Psychosocial Safety Climate Helped Alleviate Work Intensification Effects on Presenteeism during the COVID-19 Crisis? A Moderated Mediation Model

**DOI:** 10.3390/ijerph192013673

**Published:** 2022-10-21

**Authors:** Sari Mansour, Malik Faisal Azeem, Maureen Dollard, Rachael Potter

**Affiliations:** 1 School of Business Administration, TÉLUQ University of Quebec, Montreal, QC H2S 3L5, Canada; 2 Psychosocial Safety Climate Global Observatory, Justice & Society, University of South Australia, Adelaide, SA 5001, Australia; 3 School of Medicine, University of Nottingham, Nottingham NG7 2RD, UK

**Keywords:** psychosocial safety climate (PSC), work intensification, sickness presenteeism, nurses, healthcare, COVID-19

## Abstract

Healthcare sector organizations have long been facing the issue of productivity loss due to presenteeism which is affected by psychosocial safety climate (PSC) and work intensification. Presenteeism has visibly increased among nurses during COVID-19 pandemic period. Grounded in COR theory and sensemaking theory, the current study aimed to examine the role PSC plays as driver or moderator to reduce presenteeism by lessening work intensification over time and the impact of work intensification over time on presenteeism during the COVID-19 pandemic. Adopting a time-lagged research design, this study gathered data from randomly selected registered nurses, practicing in Québec, Canada in two phases, i.e., 800 at Time 1 and 344 at Time 2 through email surveys. The study results showed that (1) PSC reduces presenteeism over time by reducing work intensification at time 1; (2) PSC moderates the relationship between work intensification at time 1 and work intensification at time 2; and (3) PSC as moderator also lessens the detrimental effect of work intensification at time 2 on presenteeism at time 2. Presenteeism among nurses affects their health and psychological well-being. We find that PSC is likely an effective organizational tool particularly in crises situations, by providing an organizational mechanism to assist nurses cope (through a resource caravan, management support) with managing intensified work.

## 1. Introduction

During the COVID-19 crisis, healthcare professionals, especially nurses as front-line health care providers, face multiple mental health issues (i.e., anxiety, depression) due to stressful working conditions (e.g., high workload) [1] and a lack of adequate resources (e.g., protective equipment, clinical resources, i.e., personal protective equipment) [2]. Indeed, during the pandemic, nurses are expected to experience psychological trauma in the form of anxiety and stress due to increased patient care, longer shifts, and a lack of sleep and avoidance of breaks [3]. Increasing workloads are becoming inexorable in other types of work too [4] and essentially implicates a process of continuously escalating job demands that need to be met in a specified or short time [5,6].

Healthcare professionals were required to put in additional effort to meet the increased healthcare demands with constrained clinical resources due to the pandemic outbreak. New job demands resulting from accelerated change can be primarily illustrated as work intensification (WI) that requires an increased level of effort from an employee to be invested during the workday [5]. Persistent and chronic levels of job demands deplete energy and capacity for recovery leading to high levels of burnout [7]. High job demands may then restrict individuals’ choices leading them to be present at work even when they are ill. Job demands may become job stressors (presence-inducing situations) due to additional efforts required from the employees who endeavor to safeguard their performance in an unusual/changed scenario, see, e.g., [8]. Such an issue has become critical for nursing staff during the COVID-19 pandemic and requires intervention to protect the health and safety of healthcare professionals.

The ongoing pandemic crisis is creating multiple risks (e.g., current and future health issues, financial or job insecurity) and challenges (e.g., long work hours, increased pressures due to work-overload and staffing shortage, etc.) [9], thus increasing the potential for sickness presenteeism (PRTSM) particularly in healthcare sector. PRTSM can be defined as employees’ attendance behavior at workplace, when they do not exhibit adequate performance due to physical and/or psychological (i.e., cognitive or emotional) problems or due to being ill [8,10]. Karanika-Murray and Biron [11] advance that employees continue working even when they are sick to balance health constraints and performance demands. PRTSM intensifies in a situation where pressures to remain working during illness persist for a longer period [9] such as during pandemics, i.e., COVID-19, e.g., [12,13]. Management of PRTSM is considered a long-term investment for both individuals’ and organizational wellbeing [9] where workers are gauged through their work completion (i.e., work outcome) or ‘avoiding distraction’ (i.e., work process) [14]. PRTSM is associated with poor psychosocial environment, i.e., high job demands [8] or lack of resources [15,16,17] and has been shown to affect care quality in nursing contexts [18]. PRTSM has been reported as high as 40% of participants in a survey of 34 countries [19,20]. However, PRTSM has been less examined compared to other related health issues such absenteeism [21]. Thus, greater effort must be devoted in exploring this phenomenon [22] and in examining the less proximal work-related factors on ill-health PRTSM such as organizational climate [23].

Psychosocial safety climate (PSC) can be defined as employee’s shared perception of policies, practice and procedure regarding psychological health and safety prevention [24]. PSC includes management commitment and support to prevent stress and psychological health as well as an effective system of communication and involvement of both employees and employers in health-related issues [25,26]. PSC can be operationalized at the organizational and group level, e.g., [27] as well as at the individual level [28,29]. Such a climate is expected to contextually address psychological strain and occupational stressors in the healthcare sector [30]. PSC may act as an independent, mediator and moderator variable [31]. It predicts psychosocial work design and outcomes, e.g., [32] and moderates the effects of psychological and emotional job demands on workers depression [33]. Recently, PSC also mediated the relationship between employees’ perception of high-performance work practices and service recovery performance [34]. While Liu, Lu [23] reported that PSC directly influenced PRTSM, we believe that this relationship is likely to be indirect as PSC shapes working conditions, cf [35] and predicts psychological strain through psychological demands [32]. Thus, there exists a literature gap examining the indirect relationship between PSC and PRTSM, especially during a complex and consistently changing pandemic crises period. Cho, Sagherian [36] emphasized the need of additional support from the hospital resources (PSC in this case) and asked researchers to examine nurses’ perception about these resources to better deal with the stress during pandemic situation. According to Min, Kang [37], the relationship between WI and PRTSM needs to be examined, especially in the context of nursing [38] during these taxing times of the COVID-19 [1,15,36].

The current cross lagged study with two waves of data responds to these critical research gaps highlighted by researchers, by examining the relationships over time between PSC, WI and PRTSM during the COVID-19 pandemic. This research draws on conservation of resources [39] and sensemaking theory [40]. Christianson and Barton [41], by focusing on the three critical facets of sensemaking perspective, (i.e., noticing, meaning making, and action), highlighted the importance of this theory and unique opportunity to further use it during the COVID-19 pandemic crises.

Our study seeks to:

1. Explore the effects of WI on nurses’ PRTSM behaviors during the pandemic period [38,42,43].
2. Examine the relationship between PSC and other job demands (WI in this case) [44,45].
3. Investigate the precursor role of PSC by examining its indirect effect on PRTSM at time 2 (PRTSM T_2_) via WI at time 1 (WI T_1_).
4. Explore a moderated mediation model by testing the moderating role of PSC on the relationship: A. between WI T_1_ and WI at time 2 (WI T_2_). B. between WI T_2_ and PRTSM T_2_, C. the indirect effect of WI T_1_ on PRTSM T_2_. To our knowledge, there is no study that examined such a relationship.

Results may help healthcare organizations to minimize the job-related negative effects and to provide better working conditions in a post-COVID context. In the next section, we explain the theoretical foundation leading to our hypotheses.

**Figure 1:**
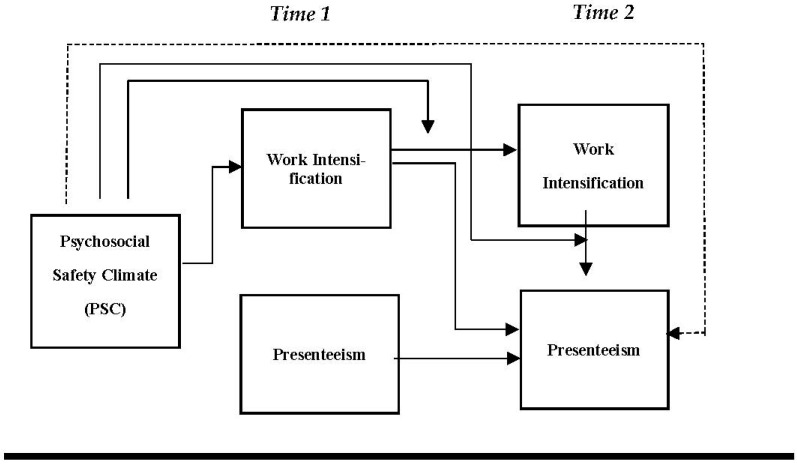
Conceptual model for Psychosocial Safety Climate (PSC), work intensification (WI) and Presenteeism (PRTSM).

Our conceptual model is presented in Figure 1.

**HYPOTHESES DEVELOPMENT**

**Work Intensification (WI) as a mediator between psychosocial safety climate (PSC) and presenteeism (PRTSM)**

The relationship between high job demands and PRTSM has been established in past cross-sectional [20] and longitudinal studies [8,46].

The PRTSM may be reduced by job resources (e.g., PSC) [23] as job resources are expected to provide nurses shield against the harmful effects of intensified work further resulting in reduced the chances of sickness. While this last study has revealed that PSC was related directly to PRTSM, it seems that the link could be indirect. Indeed, it is well known that PSC is a precursor of working conditions and is negatively associated with daily job demands and fatigue levels (i.e., long working hours) [47]. Some longitudinal studies, e.g., [27,32,48] have confirmed that PSC affected work outcomes through working conditions (e.g., demands and resources). For instance, Dollard, Opie [32] revealed a mediating effect of psychological demands between PSC and strain.

Regarding the Conservation of Resources Theory (COR) [49], energetic resource losses due to high demands may lead to drop in one’s resource reservoir to encounter new job demands and may prompt a loss spiral [50]. Wright and Hobfoll [51] indicate that individuals select coping strategies to minimize the loss of resources and to maintain their meager resources. For example, faced with loss of resources at work because of high work demands and work-family conflict, employees adopt defensive strategies such as spending less time with family to protect their resources at work [52]. In such a situation, allocating substantial energy and time to meet work constraints may lead over time to resources loss, that is, employees continue working with limited resources and no chance to recover leading to PRTSM in line with COR theory [49]. Thus, nurses while encountering high demands (e.g., WI), may decide to attend work even when they are ill to compensate their possible resource loss [53].

According to the conservation of resource theory [39], PSC may act as a resource caravan passageway [54,55,56] and can reduce the pressure of employees’ psychological and mental health by decreasing job demands [54]. The concept of ‘resource caravan passageways’, which is not well explored (Halbesleben, Neveu [57], refers to organizational ‘environmental conditions that support, foster, enrich, and protect the resources of individuals, sections or segments of workers, and organizations in total, or that detract, undermine, obstruct, or impoverish people’s or group’s resource reservoirs’ [58] (p. 119). COR’s theory though states that the resources are often beyond individuals’ control [59]. Therefore, when the level of PSC is high, nurses are expected to get more resources to cope with job demands, which in turn would decrease the level of PRTSM. Thus, the study undertakes the assumption that PSC will bring down PRTSM because they have enough resources to reduce their experience of WI. We propose the following hypothesis:

**H1.**  *Work intensification at T_1_ mediates the relationship between PSC and PRTSM at T_2_*.

**Psychosocial safety climate (PSC), work intensification (WI) and presenteeism (PRTSM): A moderated mediation model**

Nurses are expected to multitask due to time pressure to meet unpredictable demands [60]. WI is associated with time pressure and pushing workers to work faster, to do multitask and to reduce idle time [5]. Indeed, recovery from high job demands is quite critical for employees’ health and well-being and takes a period free from further job demands [61] and should be known to the employees in todays’ highly challenging work life [62]. Nevertheless, the stress response may be activated frequently and substantially in many high-demand and risky professions (e.g., healthcare organizations), as one challenge is followed by another within a relatively short time [45]. This is particularly pertinent during the crises of COVID-19 where an upward trend of longer than usual work hours has been observed among global working population resulting in severe mental health implications [47]. In line with the COR theory, people with few resources become more vulnerable to resource loss and an initial loss of resources can lead to future loss in the form of a spiral [39]. Thus, when nurses do not have the energy and are unable to recover from WI at a period during COVID-19, they may perceive another work-intensification in the future. Such prolonged loss of resources may boost the risk of consistent failure to recover adequately from work-related stress leading to a productivity loss, cf. [45]. We thus expect that WI at T_1_ (WI T_1_) would increase indirectly PRTSM at T_2_ (PRTSM T_2_) through increasing WI at T_2_ (WI T_2_). We hypothesize:

**H2.**  *WI T_2_ mediates the relationship between WI T_1_ and PRTSM T_2_*.

Managers should provide better job resources to ensure employee-wellbeing [25,63] and to shield them from strain [64]. Nurses need a safe psychosocial environment to deal with intensified work during pandemic related situation to avoid further loss of their mental and physical wellbeing. PSC can provide such a support as it plays a moderating role on the relationship between emotional demands and psychological health [54]. We can thus expect that it can moderate the longitudinal effect of WI on PRTSM. Indeed, a better understanding of commitment, support, and safety at workplace, enables nurses to change their perception about the value given to their health and safety by their employer. WI consumes valued personal resources which consequently produce psychological issues leading towards negative outcomes such as working while being sick. Sensemaking exists with the purpose of extracting meaning through interpretation of events and/or issues in somehow complex, surprising, or confusing situations [47]. Sensemaking entails the process of managing ambiguity and crisis [65] with identity threats [66]. It is considered as retrospective and ongoing social process that helps individuals construct and secure their identity by extracting cues from the shared meanings with whatever information they have [40]. These identity needs such as the sense of accomplishment and the self-efficacy are negatively affected when the work is intensified [67].

Sensemaking process plays an important role in safety climate development [68], as individuals make sense of unexpected or troublesome events using an ongoing process of action, selection, and interpretation [40]. Weick [40] also undertakes that the unusual events (e.g., crises) can disrupt sensemaking and lead to further sensemaking to create a meaningful understanding of such events. Sensemaking can happen when individuals feel that something is not right and involves shared perceptions regarding safety norms and practices by interpreting situational cues and social information, cf [69]. The provision of safety climate involves ‘shared interpretation framework’ which positively shapes individuals’ behavior and their motivation at work [68]. Such a context may pop up the sense of past (retrospective) or future (prospective) among nurses about the continuously changing environmental conditions in relation with their psychological safety. Crisis situations generally create confusion for all involved due to the occurrence of vague events that may trigger feelings of anxiety, panic, and fear, cf [70], and may consequently add into enhanced sickness PRTSM during crises situation. Such feelings of threat may provide cues to the employees to get themselves involved into sensemaking process to create situational clarity [40,71]. Nurses, during COVID-19′s crises, when they see their work intensified, initially noticed the cues from the ongoing unexpected situation, which when prolonged, put them in a complicated situation leading to continue working while sick.

As mentioned above, PRTSM may be the result of WI over time. Indeed, nurses’ perception regarding the value given to their health and safety by the employers is expected to keep changing during the pandemic situation, which may provide them with frequently conflicting cues from which to develop shared meanings from their social environment. Psychosocial safety is critical in a crisis period as it is associated with enhanced communication and knowledge sharing [72]. Such a favorable context may occur by generating cues in plausible and consistent manner. In such a complex situation, perception of having support and clear communication about health and safety by the management may enable nurses to create a sense of clarity and richness towards securing their identity, their understanding of the conflicting frames, and interpretation of their environment by focusing on plausible shared meaning. Enhanced organizational involvement, management support towards nurse’s psychological health and safety and rich communication mechanisms may play a critical role in making nurses able to extract meaning from multifaceted information during pandemic period. Therefore, nurses’ endeavor to make sense of such complex situations when they get signals from their employers regarding their psychological health and safety and get themselves involved in an ongoing situation such as accepting greater interim workload while extracting meaning of their work. Such a sensemaking processes may reduce nurses’ perception of WI and decrease PRTSM at later stages.

**Figure 2:**
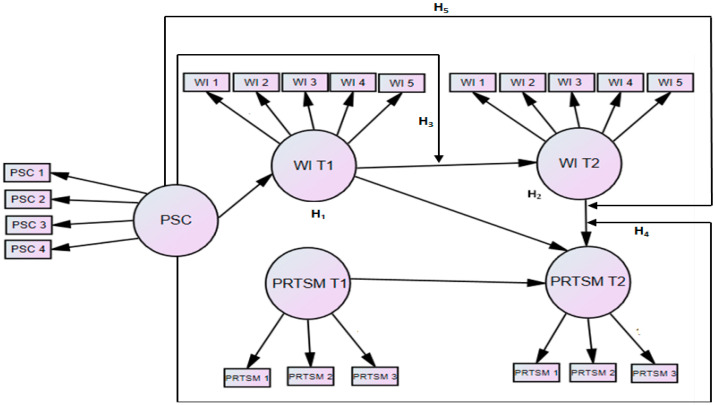
Hypothesized model showing number & direction of items, direction of the relationships and the hypotheses.

In line with the above discussion, the study also puts forth that PSC influences the positive and indirect relationship between WI T_1_ and PRTSM T_2_ (WI T_2_) which will bring a moderated mediation pattern between the said variables, exhibited in Figure 1 and Figure 2. According to Preacher and his colleagues, the moderated mediation or conditional indirect effect “occurs when the strength of an indirect effect depends on the level of some variable, or in other words, when mediation relations are contingent on the level of a moderator” [73], p. 193. Thus, the degree of the indirect effect of WI T_1_ on PRTSM T_2_ is contingent upon the nurses’ perception of PSC whether low or high. the study formulates the following hypotheses as:

**H3.**  *PSC moderates the relationship between WI T_1_ and WI T_2_, such that under the conditions of low PSC the relationship will remains positive, but under high PSC the strength of the relationship will be reduced or cancelled*.

**H4.**  *PSC moderates the relationship between WI T_2_ and PRTSM T_2_, such that under conditions of low PSC the relationship will remains positive, but under high PSC the strength of the relationship will be reduced or cancelled*.

**H5.**  *PSC moderates the conditional indirect effect of WI T_1_ on PRTSM T_2_ through WI T_2_, such that under the conditions of low PSC the relationship will remains positive, but under high PSC the strength of the relationship will be reduced or cancelled*.

## 2. Materials and Methods

### 2.1. Samples and Participants

The research project received University ethics approval (n. 2019-288). Quebec province of Canada was the most affected province of all particularly in the first wave of the COVID-19 pandemic when around 60,000 cases were reported out of which 24% cases were of healthcare professionals, cf [74]. During the COVID-19 pandemic spread, worsened work conditions particularly as significant increase in workload and work related stress were reported among nurses working in the Quebec province due to the shortage of healthcare professionals [75]. Therefore, a survey was sent by email to 2000 registered nurses in Québec selected randomly by using SPSS software from a list of 8000 members provided by professional order of nurses of Quebec. These emails were sent to each participant by the first author. The link included an introduction to the study and invited participates to the survey by requesting their consent to complete the survey on Google form on a voluntary and anonymous basis. 807 nurses responded to the survey at Time 1 (T_1_) (between October and November 2020) representing a response rate of 40.35%. Seven responses were deleted because they were nurses but worked as professors at the university. Participants were asked at T_1_ to register their email if they would like to participate at Time 2 (T_2_). We thus sent the second questionnaire to 510 valid emails. Three hundred and forty-four participants filled out the second questionnaire (response rate 67.45%) between June and July 2021. More than 91.6 % of the sample was female (coded 0) and 8.4 % were male (coded 1). Participants were found within four age categories (between 21 and 30 years (1.7%) (coded 0), between 31 and 40 (28.5%) (coded 1), between 41 and 50 (30.8%) (coded 2), between 51 and 60 (29.4%) (coded 3), and 61 and over (9.6 %) (coded 4). 80.8 % of nurses worked 12 h per day (the same shift time) (coded 0) and 19.2 % worked with rotated and variable hours from day to day (coded 1). 80.6% worked in a hospital center (coded 0), 18% worked in Affiliated University Hospital Center of Quebec (coded 1), and 11.3% worked in a hospital center for long-term care (coded 2). As for seniority, 13.4% had less than 5 years (coded 0), 12.8% had between 6 and 10 years (coded 1), 37.8% had between 11 and 20 years (coded 2), and 36% had 20 years and over (coded 3).

### 2.2. Measures

Psychosocial safety climate was assessed using the short scale of four items [35]. An example item was “Senior management show support for stress prevention through involvement and commitment”. Cronbach’s alpha (α) was 0.79 at T_1_. WI was assessed using *the intensification of job demands scale (IDS)* [5]. We reworded the five-item scale to consider the context of COVID-19. An example item was “Since the onset of COVID-19: It is increasingly rare to have enough time to complete tasks”. Cronbach’s alpha (α) was 0.88 at T_1_ and 0.85 at T_2_. We measured PRTSM by the Stanford Presenteeism Scale [14] of six items that was validated in healthcare professionals, i.e., [15] for measuring their ability to complete their tasks in a suitable manner considering PRTSM as psychosocial risk. The scale was composed of two dimensions: ‘completing work’ (items were: 1. Despite having my (health problem), I was able to finish hard tasks in my work, 2. At work, I was able to focus on achieving my goals despite my (health problem), 3. Despite having my (health problem), I felt energetic enough to complete all my work) and ‘avoiding distraction’: (items were: 1. My (health problem) distracted me from taking pleasure in my work 2. Because of my (health problem), the stresses of my job were much harder to handle; 3. I felt hopeless about finishing certain work tasks, due to my (health problem). Cronbach’s alpha (α) for ‘completing work’ was 0.79 at T_1_ and 0.86 at T_2_ and it was 0.68 for avoidance distraction at (T_1_) and 0.58 at T_2_ after removing one item because of low loading. As the value of alpha (α) for avoidance distraction was too low even after removing one item, we decided to delete this dimension from further analysis. The scale was thus considered as unidimensional latent variable with three items which were used to assess presenteeism. All items of all scales were assessed using a five points likert scale going form strongly disagree (1) to strongly agree (5). Age, type of hours (stable from day to day or variable), department and seniority were controlled.

### 2.3. Data Analysis

We proceeded in two steps to analyze the data using AMOS software version 24. Firstly, we conducted a CFA to examine the measurement model. Secondly, the research hypotheses were tested using the structural equation modelling (SEM). Maximum likelihood method was used to estimate the parameters. The chi-square/df, CFI, TLI, IFI [76] and RMSEA [77] were used to assess the fit of the measurement as well as structural model. To test the indirect effects, bootstrap analysis [78] was used which overcomes the limits of Baron and Kenny [79] approach, in particular the statistical power problem [80], and the decrease in type I error [81] and does not rely on the assumption of a normal sampling distribution [82]. Bias-corrected percentile method with two-tail significance level, 95 percent confidence intervals and 2000 bootstrapping re-samples were run. As for the moderated mediation model, we adopted the approach of Preacher et al. [82]. Indeed, one can claim moderated mediation if one of the paths in the causal system (either the X→M or M→Y path) is moderated and if the “conditional indirect effect of X on Y through M is statistically different from zero at some value(s) of the moderator but not at another value or values” [82] (p. 3). We thus tested three paths to provide evidence of our moderated mediation that is, WI T_1_ to WI T_1_ (X→M), WI T_2_ to PRTSM T_2_ (M→Y) and WI T_1_ to PRTSM T_2_ (conditional indirect effect of X on Y through M) at three values of PSC.

## 3. Results

Means, standard deviations, correlations among variables are presented in Table 1. Pearson correlation method was used.

As shown in Table 1, PSC correlates negatively with WI T_1_ and with PRTSM T_1_, but the correlations with WI T_2_ and with PRTSM T_2_ are not significant. Table 1 indicates also that WI T_1_ correlates positively with WI T_2_ and with PRTSM T_2_. Table 1 reveals that the correlation between PRTSM T_1_ and PRTSM T_2_ is positive and significant. Finally, the correlations between all our control variables and the study’s variables.

### 3.1. Results of Confirmatory Factor Analysis

#### 3.1.1. Measurement Model

The measurement model consisted of five latent factors measured at two time points: WI and PRTSM measured at T_1_ and T_2_, and PSC measured at T_1_. The results of CFA showed that the measurement model fit well the data, as fit indices were good: χ²/df = 1.926 (χ² = 308.205, df. = 160), CFI = 0.95, TLI = 0.95, IFI = 0.95 and RMSEA = 0.05. The *t*-values for all standardized factor loadings were significant as shown in Table 2.

As for discriminant validity, we compared correlations between each pair of variables with the convergent validity (AVE) of the dimension. Results indicate that the AVE of WI T_1_ (0.60) was higher that the correlations between WI T_1_ and each of WI T_2_ (0.16), PSC (−0.36), PRTSM T_1_ (0.31) and PRTSM T_2_ (0.16). This indicates that WI T_1_ is a different construct. The findings also reveal that the AVE of WI T_2_ (0.50) was higher than the correlations between WI T_2_ and each of PSC (−0.09), PRTSM T_1_ (0.15) and PRTSM T_2_ (0.19). This result confirms that WI T_2_ was a different construct. As for PSC, results show that the AVE of this variable (0.52) was higher than its correlation with PRTSM T_1_ (−0.26) and PRTSM T_2_ (−0.08) meaning that PSC was a different concept. Finally, the findings revealed that the AVE of PRTSM T_1_ (0.58) was higher than its correlation with PRTSM T_2_ (0.26) signifying that the two concepts were different.

#### 3.1.2. Structural Model

We used incomplete cross-lagged panel analyses to test all the hypotheses simultaneously. Except for PSC, which was measured only at T_1_, all other variables were measured at two pints of time. The quality of the structural model was good as indices were χ²/df = 1.365 (χ² = 603.163, df. = 442), CFI = 0.94, TLI = 0.93, IFI = 0.94 and RMSEA = 0.03. As for convergent validity of constructs (AVE), they were acceptable for all our constructs. For PSC, AVE was 0.51, for WI T_1_, AVE was 0.60; while at T_2_, AVE was 0.50. Finally, for PRTSM T_1_, AVE was 0.69 and CR was 0.87 while AVE for this construct at T_2_ was 0.58.

To test for any moderating effects, the multi-group approach was used. AMOS uses the median values of the moderator variables to constrain all parameters to be equal between the subgroups. Like Berthelsen, Ertel [83] and Alshamsi, Santos [84], we considered a score >12 to be a high level, a score between 12 and 8 to be a moderate level, and while a score equal or below 8 as a poor level. The current study compared critical ratios for differences between parameters [85] using parameter pairing to examine the differences in unstandardized coefficients for the model between each pair of groups used for the measurement of PSC. Respondents were then divided into three groups: those with a low PSC (so a high risk for health), those with a moderate PSC (so a moderate risk for health), and those with a high PSC (so a low risk for health). Indeed, one hundred and six respondents perceived PSC in their organization as low, one hundred and thirty-five perceived it as moderate and one hundred and three perceived that PSC was high.

**Figure 3:**
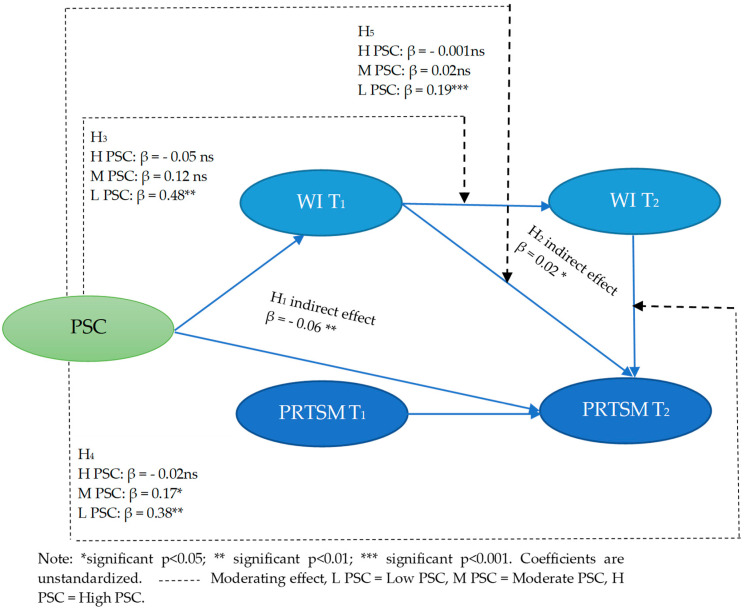
Results of indirect and moderating effects.

We proposed in hypothesis H_1_ that WI T_1_ would mediate the relationship between PSC and PRTSM at T_2_. After controlling for age, type of hours, department, seniority and PRTSM at T_1_, results shown in Table 3 and in Figure 3 reveal that this indirect effect of PSC on PRTSM at T_2_ via WI T_1_ was negative and significant (β = −0.06, *p* < 0.01, 95% Bootstrap confidence interval: (lower: 0.111, upper: −0.011). The direct effect of PSC on PRTSM at T_2_, after controlling for WI T_1_ was not significant (β = −0.03, *p* > 0.05) signifying a complete mediation. These findings support hypothesis H_1_, that is, PSC reduces PRTSM over time by reducing WI T_1_.

We predicted in hypothesis H_2_ that WI T_2_ would mediate the relationship between WI T_1_ and PRTSM T_2_. After controlling for age, type of hours, department, seniority and PRTSM at T_1_, results shown in Table 3 and in Figure 3 indicate that this indirect effect of WI T_1_ on PRTSM T_2_ via WI T_2_ was positive and significant (β = 0.023, *p* < 0.05, 95% Bootstrap confidence interval: (lower: 0.03, upper: 0.06). The direct effect of WI T_1_ on PRTSM at T_2_, after controlling for WI T_2_ was not significant (β = 0.06, *p* > 0.05) indicating a complete mediation. These findings provide evidence for hypothesis H_2_, that is, WI T_1_ increases PRTSM over time by increasing WI T_2_.

Hypothesis H3 predicted that PSC would moderate the relationship between WI T_1_ and WI T_2_, such that under conditions of low PSC the relationship will remains positive, but under high PSC the strength of the relationship will be reduced or cancelled. Findings provide support to this hypothesis. As shown in Table 4 and in Figure 3, after controlling for age, type of hours, department, seniority and PRTSM at T_1_), the results of analyses of subgroups for moderating effects indicate significant differences between the two groups, low PSC, and high PSC. Indeed, the link between WI T_1_ and WI T_2_ was statistically and significantly different between the two groups (Z = −1.98 *). We can observe that the relationship in the case of low PSC was significant and positive (β = 0.48, *p* < 0.01). This relationship becomes not significant in the case of moderate and high PSC (β = 0.12, *p* > 0.05 ns, β = −0.05, *p* > 0.05) respectively. This means that our hypothesis H_3_ is supported, that is, PSC reduces WI over time. We predicted in hypothesis H_4_ that PSC would moderate the relationship between WI T_2_ and PRTSM T_2_, such that under conditions of low PSC the relationship will remain positive, but under high PSC the strength of the relationship will be reduced or cancelled.

Table 5 and in Figure 3 also indicate that, after controlling for age, type of hours, department, seniority and PRTSM at T_1_ the effect of WI T_2_ on PRTSM T_2_ was statistically and significantly different between the two groups (low versus high PSC) (Z = –1.96 *). This effect was (β = 0.38, *p* < 0.01) in the case of low PSC and was (β = −0.02, *p* > 0.05 ns) in the case of high PSC. This signifies that PSC decreases the positive effect of WI on PRTSM over time providing support for hypothesis H4.

Coefficients are unstandardized. As for the conditional indirect effect of WI T_1_ on PRTSM T_2_ through WI T_2_, the findings shown in Table 5 and in Figure 3 reveal that, after controlling for age, type of hours, department, seniority and PRTSM at T_1_, the conditional indirect effect was positive and significant in the case of low PSC (β = 0.19, *p* < 0.001, 95% Bootstrap confidence interval: lower bounds: 0.02; upper bounds: 0.55). The same indirect effect becomes negative and not significant where the level of PSC is high (β = −0.001, *p* > 0.05 ns), 95% Bootstrap confidence interval: lower bounds: 0.02; upper bounds: 0.55. Thus, PSC moderates the mediating role of WI T_2_ between WI T_1_ and PRTSM T_2_ supporting thus our hypothesis H_5_. In sum, our findings provide support for our moderated mediation model as we found evidence of moderation for two paths (WI T_1_→WI T_2_ and WI T_2_→PRTSM T_2_) and revealed that the conditional indirect effect of WI T_1_ on PRTSM T_2_ through WI T_2_ was statistically different from zero at a high value of PSC but not at moderate or low values, cf. [73,86].

## 4. Discussion

This study is unique and the first of its kind that aims to examine nurses’ perspective of making sense of PSC and its effect on WI and PRTSM during the pandemic. We used a cross-lagged panel analysis and grounded the work in the Conservation of Resource (COR) and sensemaking (SM) perspectives. Sensemaking approach is evolving, and its assumptions do not restrict any data gathering techniques and can be used to amplify the richness of different research designs. Sensemaking being a narrative based approach primarily focuses on equivocality to understand, measure, and manage the perceived complex and ambiguous situations, also see [65,87]. Using sensemaking perspective in healthcare during the times of COVID-19 pandemic brings both conventional and unconventional reasoning of the effects of PSC on PRTSM over time. The noticing, meaning making, and action perspectives of sensemaking during COVID-19 pandemic crises, also see [41] have been complex and equivocal for the nurses due to the chaos and uncertainty triggering their feelings of anxiety, panic and fear eventually affecting their PRTSM. The current study uncovered that PSC decreases PRTSM over time during the times of COVID-19 pandemic by decreasing WI over time. Indeed, we found support for PSC as a precursor of WI leading to a decrease in PRTSM over time. Our findings also revealed another role of PSC, that is, a moderator between WI over time and the relationship between WI T_2_ and PRTSM at T_2_. Additionally, PSC moderated the conditional indirect effect of WI on PRTSM over time. This is plausible for the ongoing WI from T_1_ to T_2_ and due to the uncertainty and enhanced pressure on nurses during COVID-19, nurses are forced to expand their energies (i.e., additional resource investment) to manage the extensive workloads. Indeed, when nurses perceive a high workload, they may be afraid about the growth of COVID-19 which would intensify their workload in the future, leading them to continue working while being sick, especially with the shortages in healthcare organizations. They may create a point of reference to link their ideas with broader network by creating meaning of the events, increased communication to build narrative, retrospection, and shared understanding in a plausible manner. In such situations, PSC may decrease nurses’ PRTSM regarding their ability to manage WI over time (H_1_). In other words, a high level of PSC may lead to decrease nurses’ presence at work to complete work tasks while ill over time. Nurses’ perception of PSC as resource caravan passageway, also see [54,55,56,88] motivates them to acquire new resources (i.e., emotions, energy and time, support form managers) to fulfill their workplace requirements [39,49]. Using COR context, PSC as ‘resource caravan passageways’ helps however nurses to keep an eye on potential and actual resource loss and might let them feel able to concentrate, to manage stress and even to take some pleasure at work. This finding confirms that PSC influences PRTSM indirectly via reducing WI, which is not consistent with the study of Liu, Lu [23] who found that the relationship was direct.

The study also showed that PSC moderated WI over time (H_2_) which is in line with the findings of the past studies, e.g., [24,44,89]. Nurses due to the COVID-19 spread have been encountering WI resulting in increased psychological pressures. Nurses continuously frame and reframe the changing complex events with restricted information flow in an equivocal and plausible manner. The presence of high PSC essentially helped them create an understanding about to better deal with the intensified workloads over time (i.e., six months’ time lag in this case) during COVID-19 crises period by enabling them to address their identity needs (self-efficacy, self-enhancement, etc.) in a better way to move along the sensemaking process. The results also correspond to the conclusions of past studies where PSC as resource caravan passageway protects nurses’ resources’ depletion, e.g., [29].

This finding reflects that WI is required to be addressed reduce PRTSM. PSC moderated the relation between WI T_2_ and PRTSM T_2_. It also moderated the mediating role of WI T_2_ between WI T_1_ and PRTSM T_2_. Regarding COR theory [49], employees capitalize on available resources while being sick [53] where they conserve and create resources based on actual or threatened loss by developing new resources and by developing control over the stressful situation. In line with the COR perspective, in the presence of PSC, whether low or high, PRTSM decreased when nurses managed WI T_2_ and when they faced resource related threats (e.g., time, efforts, or energies) due to the prevailing uncertainty of COVID-19 crisis. At such, they had to continuously invest additional resources to avoid existing and potential resource losses and adopt defensive behaviors and became exhausted while dealing with the fear and anxiety.

The main implication of our findings for organizational management is that valuing the psychological and social health and safety of nurses may result in additional benefits for them and for the organization. Intervention research shows that PSC can be increased significantly within four months and can be sustained even during the COVID-19 with top management will [90]. Indeed, employees always look for management support for stress prevention [91] as employee perception of commitment or involvement of their senior management in the psychosocial work environment reduces their stress and their psychological health issues [91,92].

### Study Strengths, Limitations and Future Research

This research mainly extends literature by highlighting the need to address the phenomenon of PRTSM in relation with PSC and WI particularly in the times of COVID-19 pandemic crises. Secondly, this research is unique in terms of its context and extended theoretical considerations (i.e., Sensemaking and COR) towards the use of PSC as a factor impacting on nurses’ PRTSM over time and through addressing the issue of WI, which not only plays its role during COVID-19 pandemic period but in other crises situations as well. Thirdly, the study adds to the literature by demonstrating, using a longitudinal design, how PSC can be related to WI and PRTSM during COVID-19 pandemic outbreak and organizational turbulence. It addresses a conceptual and theoretical gap related to PRTSM.

Despite holding multiple strengths, our research does have certain limitations. In the current study, we could not test the organizational level differences regarding the impact of PSC on WI and PRTSM over time due to the confined scope of the study. This study could have incorporated other healthcare professionals as well to cross validate the results but the unavailability of doctors/physicians and other healthcare workers due to multiple issues for example, enhanced workloads and pandemic related risk factors restricted us to do so. Based on our findings, future studies should also test other critical factors like uncertainty and fear. Our study model may also be examined on other sectors particularly those that are more labor intensive, where interactions are unavoidable, and where highly risky work environments exist using different time frames to check its generalizability. Future studies could also use objective data (e.g., nurses’ attendance/sick leave records, performance reports/reviews/appraisals, overtime record, types of sickness/medication used by the employees in different time lags) to avoid potential issues of CMV/CMB. Our longitudinal design and moderation effects are not likely attributable to CMV. We also propose to test this model in the context of teams where inter and intra-team variance may be examined over time using both sensemaking and COR perspectives during the crises period in healthcare settings. According to Seddighi, Dollard [93], fear prevailing in the work environment affects employees in individual and collective capacities resulting in physical, psychological, and social damage. Future research could consider other potential mediators and moderators that may cause stress, anxiety, or fear for the employees in different situations in healthcare sector and its allied areas. Future studies could also use structural equation modeling with the PLS estimator because of its importance of being powerful, robust and reliable multivariate statistical analysis technique to deal with complex models as the one used in the current study.

## 5. Conclusions

Nurses’ PRTSM is an important issue because it is associated with employees’ mental and physical health conditions [53] and can lead to productivity loss [94]. This issue of PRTSM has become more prevalent in healthcare organizations during the pandemic of COVID-19, harming nurses’ health and psychological well-being. Our study revealed that PSC can be an excellent organizational tool to support healthcare professionals during a crisis like COVID-19, as it likely enhances resources at work and helps professionals cope with WI to reduce PRTSM. Although PSC is an antidote to WI, this stressful work condition should be avoided by building a strong PSC. In other words, when nurses perceive that organizations, managers, supervisors or assistant head nurses offer policies, procedures and practices to identify and resolve problems that threaten their well-being and psychological safety, they feel that they have more stable and safer working conditions [44]. We should however note that such a climate is not sufficient to enhance wellbeing in a complex work environment like healthcare, and organizations must implement effective human resources management practices such as training, autonomy, communication to build a safer environment. In addition, leadership style must be supportive to such initiatives to get the best result. Sense-giving as an attempt to influence employees’ meaning construction towards redefinition of organizational reality helps employees to better deal with the change and shapes up their behavior. Thus, creating an enhanced PSC makes nurses feel lesser workload related pressures and complete their job tasks during COVID-19 pandemic period.

## Figures and Tables

**Table 1:** Means, standard deviations and correlations among variables.

	M	SD	1	2	3	4	5	6	7	8	9
1. Age			1								
2. Hours’type			0.10	1							
3. Department			−0.08	−0.06	1						
4. Seniority			0.52 **	0.14 *	−0.09	1					
5. PSC	2.63	0.91	0.10	−0.07	−0.02	0.06	**0.79**				
6. WI T_1_	4.21	0.73	−0.03	0.06	0.02	−0.06	−0.31 **	**0.88**			
7. WI T_2_	2.92	1.26	−0.03	−0.01	0.03	−0.09	−0.06	0.15 **	**0.85**		
8. PRTSM T_1_	4.09	0.74	−0.01	0.04	0.05	−0.00	−0.23 **	0.25 **	0.11 *	**0.79**	
9. PRTSM T_2_	3.21	1.45	0.01	0.10	0.11 *	−0.02	−0.08	0.14 *	0.17 **	0.23 **	**0.86**

Notes: * *p* < 0.05; ** *p* < 0.01, M = mean, SD = standard deviation, Cronbach’s alpha (α) is on the diagonal and is presented in bold.

**Table 2:** Items loading and convergent validity (AVE).

Variables	Items	Loading	AVE
WI T_1_	Since the COVID-Crisis:		0.60
	1. It is increasingly rare to have enough time for work tasks	0.80	
	2. It is increasingly harder to take time for breaks.	0.78	
	3.The time between the more intense work phases has decreased.	0.84	
	4. One has more often to do two or three things at once (such as eating lunch, writing emails, and talking on the phone).	0.80	
	5. Ever more work has to be completed by fewer and fewer employees	0.63	
WI T_2_	Since the COVID-Crisis:		0.50
	1. It is increasingly rare to have enough time for work tasks	0.74	
	2. It is increasingly harder to take time for breaks.	0.83	
	3.The time between the more intense work phases has decreased.	0.66	
	4. One has more often to do two or three things at once (such as eating lunch, writing emails, and talking on the phone).	0.72	
	5. Ever more work has to be completed by fewer and fewer employees	0.57	
PSC	1. Senior management shows support for stress prevention through involvement and commitment.	0.81	0.52
	2. Senior management considers employee psychological health to be as important as productivity.	0.53	
	3. There is good communication here about psychological safety issues which affect me.	0.89	
	4. In my organization, the prevention of stress involves all levels of the organization.	0.59	
PRTSM T_1_	1. Despite having my (health problem), I was able to finish hard tasks in my work.	0.67	0.58
	2. At work, I was able to focus on achieving my goals despite my (health problem).	0.88	
	3. Despite having my (health problem), I felt energetic enough to complete all my work.	0.72	
PRTSM T_2_	1. Despite having my (health problem), I was able to finish hard tasks in my work.	0.69	0.69
	2. At work, I was able to focus on achieving my goals despite my (health problem).	0.94	
	3. Despite having my (health problem), I felt energetic enough to complete all my work.	0.84	

Note: AVE = Average variance extracted.

**Table 3:** Indirect effects: Test of mediation models (hypothesis H1 and H2).

Path	β	*p*-Value	Bootstrapping Bias-Corrected Percentile Method 95% CI	
			Lower	Upper
PSC on PRTSM T_2_ via WI T_1_	−0.06	<0.01	0.11	−0.01
WI T_1_ on PRTSM T_2_ via WI T_2_	0.02	<0.02	0.03	0.06

Note: Coefficients are standardized.

**Table 4:** Multigroup analysis: Test of the moderated mediation model (hypothesis H3, H4, H5).

Independent Variables	Dependent Variable: WI T_2_ (X→M, H_3_)					
	Low PSC (≤8, High Risk)		Moderate PSC (>8–12, Moderate Risk)		High PSC (Low Risk, >12, Low Risk)	
	Β	*CR*	Β	*CR*	Β	*CR*
1. Age	−0.001 ns	−0.01 ns	−0.06 ns	−0.51	0.36 ns	0.22
2. Hours’type	0.04 ns	0.18	0.04 ns	0.15	0.11 ns	−0.23
3. Department	−0.20 ns	−1.5	0.12 ns	0.78	−0.16 ns	−0.72
4. Seniority	−0.13 ns	−1.65	−0.07 ns	−0.75	−0.05 ns	−0.50
5. WI T_1_	0.48 **	2.77	0.12 ns	0.70	0.05 ns	0.31
R²	0.17 **		0.02	0.08		
**Independent Variables**	**Dependent Variable: PRSTSM T_2_ (M→Y, H_4_** **)**					
1. Age	−0.20 ns	1.27	−0.13 ns	−1.33	0.20 ns	1.37
2. Type of hours	0.20 ns	0.60	0.37 *	1.92	0.31 ns	0.79
3. Department	0.07 ns	0.36	0.15 ns	1.23	0.02 ns	0.11
4. Seniority	−0.001 ns	−0.01	−0.02 ns	−0.24	−0.02 ns	−0.22
5. PRSTSM T_1_	0.45 **	2.64	0.29 *	2.28	0.45 ns	2.64
6. WI T_2_	0.38 **	2.11	0.17 *	1.95	−0.02 ns	−0.24
R²	0.15 *		0.14		0.11	

Note: * significant *p* < 0.05; ** significant *p* < 0.01.

**Table 5:** Multi-group analysis of PSC: Conditional indirect effect of WI T_1_ on PRTSM T_1_ through WI T_2_ (X on Y through M, H5).

Low PSC (≤8, High Risk)			Moderate PSC (>8–12, Moderate Risk)			High PSC (Low Risk, >12, Low Risk)		
β	Bootstrapping Bias-corrected percentile method 95% CI		β	Bootstrapping Bias-corrected percentile method 95% CI		β	Bootstrapping Bias-corrected percentile method 95% CI	
	Lower	Upper		Lower	Upper		Lower	Upper
0.19 ***	0.02	0.55	0.02 ns	−0.03	0.12	−0.001 ns	0.06	0.00

Note: *** significant *p* < 0.001.

## Data Availability

The data presented in this study are available on request from the corresponding author. The data are not publicly available due to privacy.
